# The SREBP-dependent regulation of cyclin D1 coordinates cell proliferation and lipid synthesis

**DOI:** 10.3389/fonc.2022.942386

**Published:** 2022-08-24

**Authors:** Arwa Aldaalis, Maria T. Bengoechea-Alonso, Johan Ericsson

**Affiliations:** ^1^ Division of Biological and Biomedical Sciences, College of Health and Life Sciences, Hamad Bin Khalifa University, Education City, Doha, Qatar; ^2^ School of Medicine and Medical Science, University College Dublin, Dublin, Ireland

**Keywords:** srebp, cyclin D1, proliferation, cancer, lipids, CDK4, CDK6, Rb

## Abstract

The sterol regulatory-element binding protein (SREBP) family of transcription factors regulates cholesterol, fatty acid, and triglyceride synthesis and metabolism. However, they are also targeted by the ubiquitin ligase Fbw7, a major tumor suppressor, suggesting that they could regulate cell growth. Indeed, enhanced lipid synthesis is a hallmark of many human tumors. Thus, the SREBP pathway has recently emerged as a potential target for cancer therapy. We have previously demonstrated that one of these transcription factors, SREBP1, is stabilized and remains associated with target promoters during mitosis, suggesting that the expression of these target genes could be important as cells enter G1 and transcription is restored. Activation of cyclin D-cdk4/6 complexes is critical for the phosphorylation and inactivation of the retinoblastoma protein (Rb) family of transcriptional repressors and progression through the G1 phase of the cell cycle. Importantly, the cyclin D-cdk4/6-Rb regulatory axis is frequently dysregulated in human cancer. In the current manuscript, we demonstrate that SREBP1 activates the expression of cyclin D1, a coactivator of cdk4 and cdk6, by binding to an E-box in the cyclin D1 promoter. Consequently, inactivation of SREBP1 in human liver and breast cancer cell lines reduces the expression of cyclin D1 and attenuates Rb phosphorylation. Rb phosphorylation in these cells can be rescued by restoring cyclin D1 expression. On the other hand, expression of active SREBP1 induced the expression of cyclin D1 and increased the phosphorylation of Rb in a manner dependent on cyclin D1 and cdk4/6 activity. Inactivation of SREBP1 resulted in reduced expression of cyclin D1, attenuated phosphorylation of Rb, and reduced proliferation. Inactivation of SREBP1 also reduced the insulin-dependent regulation of the cyclin D1 gene. At the same time, SREBP1 is known to play an important role in supporting lipid synthesis in cancer cells. Thus, we propose that the SREBP1-dependent regulation of cyclin D1 coordinates cell proliferation with the enhanced lipid synthesis required to support cell growth.

## Introduction

Cell cycle progression, and thereby cell proliferation, is controlled by the sequential activation and inactivation of specific cyclin–cdk complexes. In G1, the sequential activation of cdk4/6-cyclin D and cdk2-cyclin A/E complexes ensures complete phosphorylation and inactivation of the Rb family of transcriptional repressors, thereby activating E2F transcription factors and inducing the expression of their proliferative target genes (1). Many human tumors display a defect in Rb function, e.g., loss of Rb or amplification of specific cyclin or cdk genes (2–4). The cell cycle is initiated by the activation of cdk4/6 by the formation of cyclin D-cdk4/6 complexes (1). There are three separate cyclin D genes, namely, cyclin D1, D2, and D3, that display cell- and tissue-specific expression. Although all D-type cyclins activate cdk4/6 and Rb phosphorylation, overactivation of cyclin D1 is far more common in human tumors when compared to D2 and D3 (5). Thus, the expression of cyclin D1 needs to be tightly controlled. A number of growth-promoting signaling pathways and oncogenes activate the cyclin D1 gene (5). In addition, the cyclin D1 protein is also tightly controlled, especially its proteasome-mediated degradation. Cyclin D1 has a short half-life, and several ubiquitin ligases have been put forward as potential regulators of cyclin D1 degradation (6). Although the identity of the ligase(s) responsible remained elusive, it was clearly demonstrated that phosphorylation of cyclin D1 by GSK-3 was important for its degradation (7, 8). However, a series of recent reports identified Ambra1, a cullin 4-associated E3 ligase, as being involved in the degradation of cyclin D1 (9–11).

Since cdk4/6-cyclin D complexes are required for cells to enter the cell cycle, inhibitors of these complexes have emerged as potential anticancer treatments (1, 12, 13). Cdk4/6 inhibitors, i.e., palbociclib, ribociclib, and abemaciclib, have demonstrated promising results in clinical trials for patients with hormone-receptor-positive breast cancers. As a result, these compounds are currently being evaluated in more than 300 clinical trials for over 50 tumor types (1). Unfortunately, the development of drug resistance has been reported following cdk4/6 inhibitor treatment (1, 12). A number of hypotheses have been put forward to explain the development of cdk4/6 inhibitor resistance (1, 12). A recent study demonstrated that prolonged treatment of breast cancer cells with these inhibitors resulted in the degradation of Rb and the transcriptional activation of the cdk6 gene (14). Taken together, these events promote resistance to cdk4/6 inhibitors. Importantly, the authors demonstrated that both of these events were dependent on CK1ϵ and that combining cdk4/6 inhibitors with CK1 inhibitors dramatically increased their ability to reduce tumor growth in animal models.

The sterol-regulatory-element binding protein (SREBP) pathway controls the activation of the transcription factors SREBP1a, SREBP1c, and SREBP2 (15, 16). Once activated, these transcription factors control the expression of genes associated with cholesterol, fatty acid, and triglyceride synthesis and metabolism (17–19). SREBP1c and SREBP2 are expressed in most tissues, while SREBP1a is restricted to rapidly dividing cells, such as certain immune cells (20–22). Importantly, SREBP1a is the dominant SREBP1 protein in most cancer cells and tumors. The shift from SREBP1c to SREBP1a is important, since the latter is a stronger transcription factor and it is capable of inducing all SREBP-responsive genes described to date. On the other hand, SREBP1c is mainly regulating genes involved in fatty acid synthesis, while SREBP2 is mainly regulating genes associated with cholesterol synthesis and metabolism (16, 17). All three proteins are activated in response to low levels of intracellular cholesterol. SREBP1c is also activated downstream of insulin signaling, especially in the liver (16, 17). The most clinically important SREBP target is the LDL receptor gene, since this receptor is responsible for clearing harmful LDL cholesterol from the circulation, thereby preventing atherosclerosis and cardiovascular disease. The statin family of cholesterol-lowering drugs inhibit the rate-limiting enzyme involved in cholesterol synthesis, thereby activating the SREBP pathway, resulting in the transcriptional activation of the LDL receptor, which in turn results in more LDL-receptor molecules being expressed at the plasma membrane of hepatocytes and therefore enhanced removal of LDL particles from the circulation (16). There are two forms of SREBP molecules, precursors, and mature proteins. The precursor molecules reside in the ER membrane and are transcriptionally inactive (17). However, when the levels of cholesterol in the ER membrane decline below a certain point, the SREBPs are transported from the ER to the Golgi where they are sequentially cleaved by two proteases, generating an N-terminal fragment that is released into the cytoplasm. The N-terminal fragment contains a transcriptional activation domain, a DNA-binding domain, and a C-terminal regulatory domain, primarily involved in regulating the turnover of the active transcription factors (15). The cleaved fragments are transported to the nucleus where they bind to target promoters and activate the expression of the corresponding genes. The N-terminal fragment is usually referred to as mature, nuclear, or active SREBP. In the case of SREBP1c, the same process is initiated in response to insulin signaling. However, insulin also activates the expression of the SREBP1c gene (23, 24).

Like many transcription factors, the active SREBPs are unstable and are degraded by the ubiquitin-proteasome system (25–28). We have demonstrated that nuclear SREBP1 and SREBP2 are targeted for degradation by the E3 ubiquitin ligase Fbw7 and that this requires the phosphorylation of the SREBPs by GSK-3 (29), the same kinase that targets cyclin D1 for degradation. Consequently, inactivation of Fbw7 in cells results in the accumulation of transcriptionally active versions of both SREBP1 and SREBP2 and elevated expression of SREBP target genes. This process is inhibited in response to insulin signaling as a result of AKT-mediated inactivation of GSK-3 (29, 30), and we have proposed that this could contribute to the accumulation of nuclear SREBP1c seen in response insulin treatment. Many Fbw7 targets are growth-promoting molecules, including c-Myc, c-Jun, and cyclin E, and loss of function mutations in the Fbw7 gene is common in human tumors (31, 32). The stabilization of active SREBP1a molecules in such tumors would support their rapid proliferation by enhancing the expression of genes involved in lipid synthesis. The growing interest in cancer metabolism has renewed the interest in the functional importance of lipid synthesis for cell proliferation. Thus, several studies have explored the role of SREBP-dependent lipid synthesis during cancer cell growth, both *in vitro* and *in vivo*. Because of the dominant role played by SREBP1a in cancer cells, most of these studies have focused on SREBP1. Taken together, these studies suggest that the SREBP pathway supports the enhanced demand for *de novo* lipogenesis in rapidly dividing cells (33–36). In addition, it has been demonstrated that SREBP-dependent lipid metabolism is induced in cancer cells through the PI3K/AKT/mTOR axis downstream of growth factor signaling (37–41). Importantly, SREBP1 is activated in response to transformation of cells with oncogenic mutants of PI3K and Ras (42). Inactivation of SREBP1 in cancer cells attenuates cell proliferation, both *in vitro* and in mouse tumor models (37, 38, 42–47). Apart from supporting enhanced lipid synthesis, SREBP1 may also protect cancer cells from metabolic stress and lipotoxicity by regulating the ratio between saturated and unsaturated fatty acids by controlling the expression of the fatty acid desaturase SCD1 (45, 47, 48). Thus, the SREBP pathway and the pathways that it controls have emerged as potential targets for cancer therapeutics (34, 49, 50).

Although the SREBPs are degraded by Fbw7 during interphase, active SREBP1 molecules are hyperphosphorylated and stabilized during mitosis, at least in cancer cell lines (43, 51). In fact, SREBP1 is protected from Fbw7-mediated degradation during mitosis by a mechanism involving its sequential phosphorylation by cdk1 and plk1 (44). Interestingly, we also found that SREBP1 remained associated with some of its target promoters during mitosis, and we proposed that this could be involved in a process known as mitotic bookmarking (51), where transcription factors remain associated with target genes during cell division to ensure that the corresponding genes are transcribed as cells leave mitosis (52). The aim of the current study was to identify such genes that could play important roles during early G1.

In the current study, we found that the expression of cyclin D1 is regulated by the SREBP pathway. We reported that SREBP1 binds to an E-box in the promoter of the human cyclin D1 gene, both *in vitro* and *in vivo*. Importantly, the recruitment of endogenous SREBP1 to the cyclin D1 promoter was enhanced in response to insulin stimulation, a well-established mitogen and activator of the SREBP pathway. Inactivation of both SREBP1 and SREBP2 reduced the expression of cyclin D1 in HepG2 and MCF7 cells, while expression of the active forms of the transcription factors induced the expression of cyclin D1. The changes in cyclin D1 expression under these conditions were mimicked by changes in the phosphorylation of Rb. Consequently, inactivation of SREBP1 resulted in the reduced expression of cyclin D1, reduced phosphorylation of Rb, and a partial G1 cell cycle arrest. These effects were reversed by the expression of exogenous cyclin D1. Thus, we propose that the SREBP-dependent regulation of cyclin D1 helps coordinate cell cycle progression with increased lipid synthesis to support cancer cell growth.

## Materials and methods

### Cell culture and treatments

HepG2, MCF7, and HEK293 cells were from American Type Culture Collection (ATCC). All cell culture media and reagents were from Gibco. Unless otherwise stated, HepG2 cells were grown in Minimum Essential Medium (MEM) supplemented with 10% fetal bovine serum (FBS), non-essential amino acids, sodium pyruvate, Glutamax, and antibiotic–antimycotic. The other cell lines were grown in Dulbecco’s modified Eagle medium (DMEM) supplemented with 10% FBS and all the reagents above. To arrest cells at G0/G1, cells were washed with phosphate-buffered saline (PBS) and shifted to starvation media containing 0.5% FBS for at least 24 h. To release cells from this arrest, the media was supplemented with FBS (10%).

### Lentivirus production and transduction

HEK293 cells were used to produce lentiviruses expressing the active nuclear SREBP isoforms or shRNAs. Twelve micrograms of lentiviral DNA constructs was co-transfected with lentivirus packaging mix (Dharmacon, TLP4606) by calcium phosphate precipitation, and the cells were kept in the incubator for 48 h. Afterwards, the media were collected and filtered through 0.45-μm syringe filters, and the viruses were stored in aliquots at −80°C. Cells were transduced in regular media supplemented with polybrene (8 μg/ml). Twenty-four hours later, puromycin was added at 5 μg/ml, and the selection continued for 3–4 days.

### Cell lysis and immunoblotting

Cells were lysed in buffer A [50 mM HEPES (pH 7.2), 150 mM NaCl, 1 mM EDTA, 20 mM NaF, 2 mM sodium orthovanadate, 10 mM β-glycerophosphate, 1% (w/v) Triton X-100, 10% (w/v) glycerol, 1 mM phenylmethylsulfonyl fluoride, 10 mM sodium butyrate, 1% aprotinin, 0.1% SDS, and 0.5% sodium deoxycholate] and cleared by centrifugation. For DNA pull-down (DNAP) and electromobility shift assays (EMSAs), cell lysates were prepared in the absence of SDS and sodium deoxycholate. Cell lysates were resolved by sodium dodecyl sulfate–polyacrylamide gel electrophoresis (SDS-PAGE) and transferred to nitrocellulose membranes (Millipore). To ensure that equal amounts of protein were loaded in each well, the levels of β-actin in the samples were estimated by Western blotting.

### Reagents and antibodies

Anti-FLAG antibody (M5), mouse anti-actin (A5441), and standard chemicals were from Sigma. Palbociclib was from Selleckchem (S1116). Rabbit anti-cyclin D1 was from Abcam (ab134175). Rabbit anti-SREBP1 (H-160), mouse anti-SREBP2 (1C6), mouse anti-RB (IF8) and mouse anti-HMGCS (C8) were from Santa Cruz Biotechnology. Rabbit anti-pRB(S780) (#9307) was from Cell Signaling Technology. Horseradish-peroxidase-conjugated anti-mouse and anti-rabbit IgG were from Invitrogen.

### Plasmids and DNA transfections

The human cyclin D1 promoter in pGL3Basic was a gift from Frank McCormick (Addgene plasmid #32727). pHAGE-CCND1 was a gift from Gordon Mills and Kenneth Scott (Addgene plasmid #116721). The expression vectors for FLAG-tagged nuclear SREBP1a and 6xmyc-tagged nuclear SREBP1a, SREBP1c, and SREBP2 have been described previously (29, 30, 43, 51). To generate the lentiviral expression vectors for the active SREBPs, the corresponding cDNAs were subcloned into pLKO-puro FLAG SREBP1, a gift from David Sabatini (Addgene plasmid #32017). The lentiviral cyclin D1, SREBP1, and SREBP2 shRNA vectors were obtained from VectorBuilder. Mutations in the cyclin D1 promoter construct were generated by site-directed mutagenesis. To delete the E-box, the following primers were used: forward, 5’ GTT CTT GCA ATT TAT TAA TGA AAA TGA AAG AAG ATG CAG TCG CTG AG 3’, and reverse, 5’ CAT TTT CAT TAA TAA ATT GCA AGA ACT AAT TTA GCA TGC AAG GAC GGG G 3’. To delete the putative SRE site, the following primers were used: forward, 5’ AGA GCC ACC TCC CCC TAA ATC CCG GGG GAC CCA CTC GAG GCG GAC GG 3’, and reverse, 5’ GAT TTA GGG GGA GGT GGC TCT GCA GTA GGG GAC AAC TAG GAA GGC CGG C 3’. Transfections were performed by calcium phosphate precipitation.

### RNA extraction and qPCR

RNA was extracted using Thermo GeneJet RNA Purification Kit (K0731). cDNA was generated using Applied Biosystems High-Capacity cDNA Reverse Transcription Kit. For qPCR, PowerUp SYBR Green Master Mix was used (Applied Biosystems). The primers designed to amplify target genes are provided in the table below.

**Table:** 

Gene	Forward	Reverse
**cyclin D1**	TCG TTG CCC TCT GTG CCA CA	AGG CAG TCC GGG TCA CAC TT
**GAPDH**	CCC TTC ATT GAC CTC AAC TAC A	CTG GAA GAT GGT GAT GGG ATT
**FATTY ACID SYNTHASE (FAS)**	GAA ACT GCA GGA GCT GTC	CAC GGA GTT GAG CCG CAT
**HMG-CoA Reductase**	TAC CAT GTC AGG GGT ACG TC	CAA GCC TAG AGA CAT AAT CATC
**SREBP1a**	TCA GCG AGG CGG CTT TGG AGC AG	CAT GTC TTC GAT GTC GGT CAG
**SREBP1c**	GGA GGG GTA GGG CCA ACG GCC T	CAT GTC TTC GAA AGT GCA ATC C
**SREBP2**	CTG AAG CTG GCA AAT CAA AAG	TCA TCC AAT AGA GGG CTT CCT

### Luciferase and β-galactosidase assays

Cells were transiently transfected with the indicated promoter–reporter genes in the absence or presence of the indicated expression vectors and/or shRNA. Luciferase activities were determined in duplicate samples as described by the manufacturer (Promega). Cells were also transfected with the β-galactosidase gene as an internal control for transfection efficiency. Luciferase values (relative light units, RLUs) were calculated by dividing the luciferase activity by the β-galactosidase activity. The data represent the average −/+ SEM of at least three independent experiments performed in duplicates.

### Chromatin immunoprecipitation

Chromatin immunoprecipitation was performed using the CUT&RUN Assay Kit from Cell Signaling Technology (86652) following the protocol recommended by the manufacturer. MCF7 cells were starved overnight with 0.5% FBS-containing media and either left untreated or induced with insulin for 2 h. The antibodies used for immunoprecipitation was SREBP1 (H160) and the negative control rabbit (DA1E) IgG XP isotype control antibody included in the kit. After DNA purification, a PCR reaction was performed to amplify regions around either the E-box site (forward, 5’ CCC CGT CCT TGC ATG CTA AAT 3’; reverse, 5’ CCA AAG AAT CTC AGC GAC TGC A 3’) or the putative SRE site (forward, 5’ CCG GCC TTC CTA GTT GTC CC 3’; reverse, 5’ CCC GTC CGC CTC GAG TG 3’). The PCR products were separated on 10% polyacrylamide gels in 0.5× Tris–borate–EDTA (TBE) buffer, stained with SYBR Safe and visualized on an aright CL1500 Imaging System (Invitrogen). The intensity of individual bands was quantified using the iBright Analysis Software (Invitrogen).

### DNA pull-down assays

Biotin-labeled DNA probes corresponding to either wild-type (WT) or deletion mutants of the cyclin D1 promoter region were prepared by PCR then purified. The following primers were used: forward, 5’ biotin-CTA GCA AAA TAG GCT GTC CC 3’, and reverse, 5’ CTT TAT GTT TTT GGC GTC TTC CA 3’. The template was the pGL3Basic-cyclin D1 promoter construct mentioned above. 6xMyc-tagged SREBP isoforms were overexpressed in HEK293 cells, and cell lysates were prepared in the absence of SDS and sodium deoxycholate. Lysates were pre-cleared with Protein A agarose beads; then, 90 μl of the lysates was used for the pull-down assays with 200 ng of probes. The reaction also contained 20 mM MgCl_2_ and 1 μg of sheared salmon sperm DNA. The reaction mixture was kept in an end-over-end mixer at 4°C for 1 h, followed by the addition of streptavidin agarose beads and returned to the end-over-end mixer for another 30 min. Subsequently, the reactions were spun down, the supernatants were discarded, and the beads were washed four times with cold Buffer A. Finally, protein loading buffer was added to the beads. The reactions were boiled and separated on SDS-PAGE gels and transferred to nitrocellulose membranes (Millipore). Western blots were performed with mouse anti-Myc (sc-40, Santa Cruz Biotechnology) to detect all isoforms at the same time.

### Electrophoresis mobility shift assay

FLAG-tagged SREBP1a was overexpressed in HEK293 cells, and the protein was purified using anti-FLAG agarose beads (Sigma). DNA probes corresponding to wild type or the E-box deletion of the cyclin D1 promoter were prepared by PCR and purified. The following primers were used: forward, 5’ CCC CGT CCT TGC ATG CTA AAT 3’; reverse, 5’ CCA AAG AAT CTC AGC GAC TGC A 3’. The template was the pGL3Basic-cyclin D1 promoter construct mentioned above. The 10× binding buffer contains 200 mM Tris–HCl pH 7.5 and 500 mM NaCl. The final reaction mixture contains 2 μl 10× binding buffer, 20% glycerol, 1 μg sheared salmon sperm DNA, 1 mM MgCl_2_, 1 mM dithiothreitol (DTT), and 0.5 μg bovine serum albumin (BSA). Where indicated, 500 ng of probes and 4 μl of purified FLAG-SREBP1a were added to the reactions. To generate a supershift, anti-FLAG antibody (M5) was used. The electrophoresis mobility shift assay (EMSA) reactions were separated on 4% polyacrylamide gels with 0.5× TBE buffer, stained with SYBR Safe, and visualized on an iBright CL1500 Imaging System (Invitrogen).

### BrdU incorporation assay and cell growth assay

For the BrdU incorporation assay, the BrdU Cell Proliferation ELISA Kit (abcam #ab126556) was used. Wild-type or SREBP-deficient MCF7 cells were plated in 96-well plates and incubated with the BrdU reagent overnight. The colorimetric reaction was performed as suggested by the manufacturer, and the absorbance was measured at 450 nm on a Spark Multimode Microplate Reader (Tecan). The data represent the average −/+ SEM of three independent experiments performed in triplicates. For the cell growth assay, wild-type or SREBP-deficient MCF7 cells were plated in 12-well plates. Cells were collected every 24 h and counted using the trypan blue exclusion method on a TC20 automated cell counter (Bio-Rad).

### FACS analysis

For FACS analysis, cells were harvested by treatment with trypsin, washed in phosphate-buffered saline, and resuspended in 70% ethanol for storage at −20°C until further analysis. Cells were stained with propidium iodide (70 μg/ml), and their DNA content was analyzed on a BD Accuri C6 Plus Flow Cytometer (BD Biosciences).

### Data analysis

Statistical data analyses were conducted using the GraphPad Prism 8 software. One-way ANOVA and paired t-tests were applied as indicated in the figure legends. Standard error of mean (SEM) was calculated for experimental replicates, with statistical significance set to p < 0.05.

## Results

### Members of the SREBP family of transcription factors regulate the expression of cyclin D1

**Figure 1:**
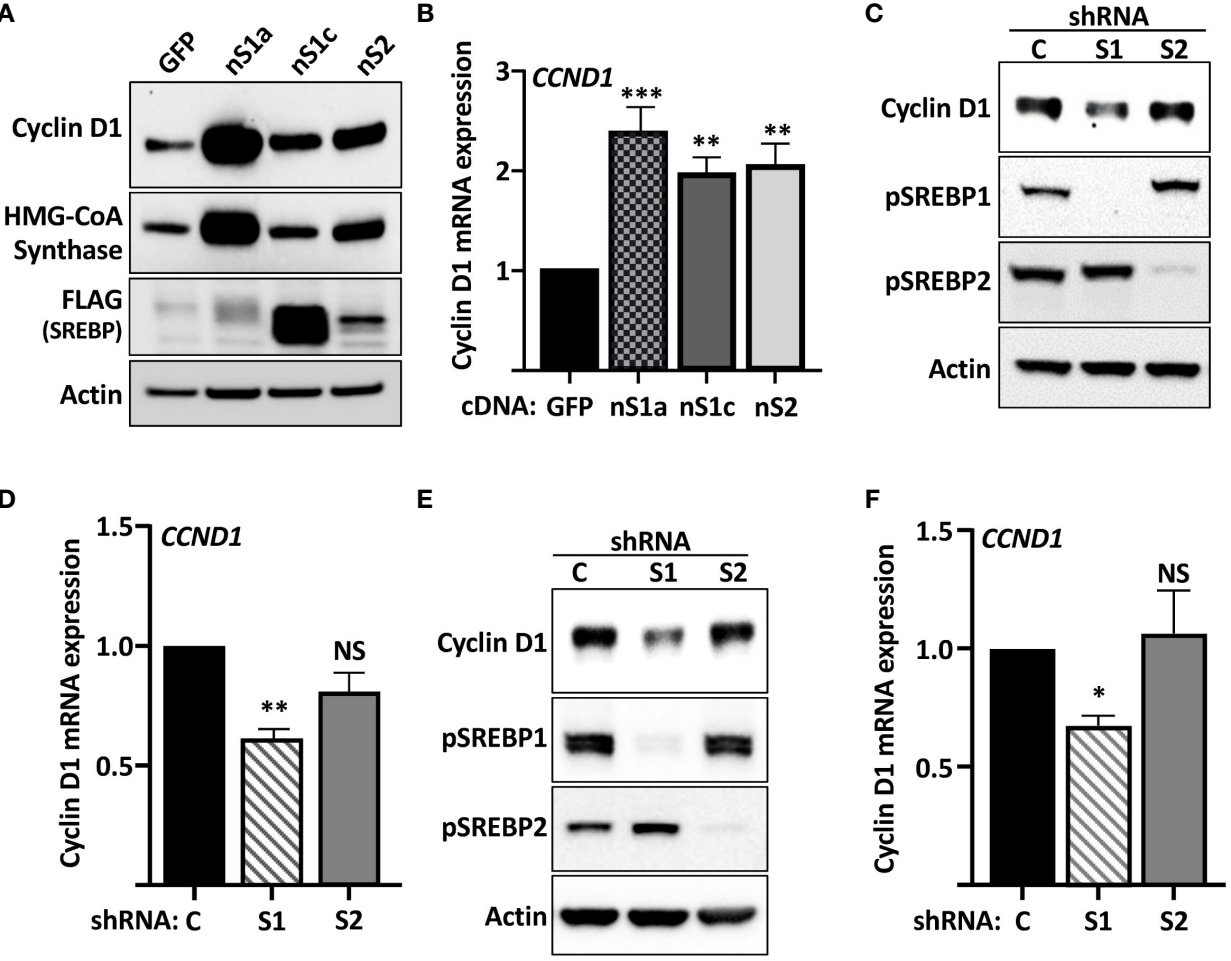
Cyclin D1 is regulated by members of the SREBP family of transcription factors. **(A)** HepG2 cells were transduced with lentiviruses encoding GFP or FLAG-tagged versions of the nuclear forms of SREBP1a (*nS1a*), SREBP1c (*nS1c*), or SREBP2 (*nS2*). The levels of cyclin D1, HMG-CoA synthase, the nuclear forms of SREBP1a, SREBP1c and SREBP2 (*FLAG*), and β-actin in total lysates were determined by Western blotting. The quantifications of cyclin D1 and HMG-CoA synthase across three independent experiments are included in **Supplementary Figure S1**. **(B)** HepG2 cells were transduced as in *(A)*, and mRNA was isolated and used to generate cDNA. The expression of cyclin D1 (*CCND1*) was determined by real-time qPCR using GAPDH for normalization. **(C)** HepG2 cells were transduced with lentiviruses encoding shRNAs, either non-targeted *(C)* or targeting SREBP1 (*S1*) or SREBP2 (*S2*). The levels of cyclin D1 and the precursor forms of SREBP1 (*pSREBP1*) and SREBP2 (*pSREBP2*) were detected by Western blotting. β-Actin was used as a loading control. **(D)** HepG2 cells were transduced with shRNA as in *(C)* and the expression of cyclin D1 was determined by real-time qPCR. **(E)** MCF7 cells were transduced with shRNA as in *(C)*, and the levels of cyclin D1 and the precursor forms of SREBP1 (*pSREBP1*) and SREBP2 (*pSREBP2*) were detected by Western blotting. **(F)** MCF7 cells were transduced with shRNA as in *(C)*, and the expression of cyclin D1 was determined by real-time qPCR. Significance was determined by one-way ANOVA with Tukey’s multiple comparisons adjustment *(B, D, and F)*. p-values lower than 0.05 were considered statistically significant. *p < 0.05, **p < 0.01, ***p < 0.001, and ****p < 0.0001. *NS*, not significant.

The gradual phosphorylation of Rb by sequential cyclin–cdk complexes is required for cells to re-enter the cell cycle and progress through G1 and into S phase. Thus, the cyclin D-cdk4/6-Rb axis could be a potential SREBP1 target. To test this hypothesis, HepG2 cells, a human hepatoma cell line, was transduced with lentiviruses expressing the nuclear versions of SREBP1a, SREBP1c, and SREBP2. As illustrated in **Figure 1A**, the expression of cyclin D1 protein was enhanced in cells expressing all three SREBP proteins, with the most significant induction observed in cells expressing SREBP1a. The induction of the well-established SREBP target gene HMG-CoA synthase displayed the same response as cyclin D1 (**Figure 1A**, quantification in **Supplementary Figure 1**). To determine if the SREBPs affected the expression of the cyclin D1 gene, the experiment was repeated followed by mRNA isolation and RT-qPCR assays. Again, all three SREBP proteins enhanced the expression of cyclin D1 (**Figure 1B**) and more traditional SREBP target genes (HMG-CoA reductase and fatty acid synthase, **Supplementary Figure S2**).

To determine if endogenous SREBP1 and/or SREBP2 could regulate the expression of cyclin D1, HepG2 cells were transduced with lentiviral shRNA constructs targeting either SREBP1 (both SREBP1a and SREBP1c) or SREBP2. Knockdown of SREBP1 resulted in a reduction in the expression of cyclin D1 protein, while inactivation of SREBP2 had no effect (**Figure 1C**). Inactivation of SREBP1 in HepG2 cells also resulted in a reduction in cyclin D1 mRNA, while the effect of SREBP2 inactivation failed to reach significance (**Figure 1D**). Inactivation of SREBP1 in human MCF7 breast cancer cells also reduced the expression of cyclin D1 protein (**Figure 1E**). As in HepG2 cells, inactivation of SREBP2 failed to affect the expression of cyclin D1 protein in MCF7 cells. This was supported by the observation that only inactivation of SREBP1 reduced the mRNA levels of cyclin D1 in these cells (**Figure 1F**). The knockdown efficiencies of SREBP1a, SREBP1c, and SREBP2 in both cell lines were monitored by RT-qPCR (**Supplementary Figure 3**). Taken together, these data suggest that SREBP1 regulates the expression of cyclin D1 in HepG2 and MCF7 cells.

**Figure 2:**
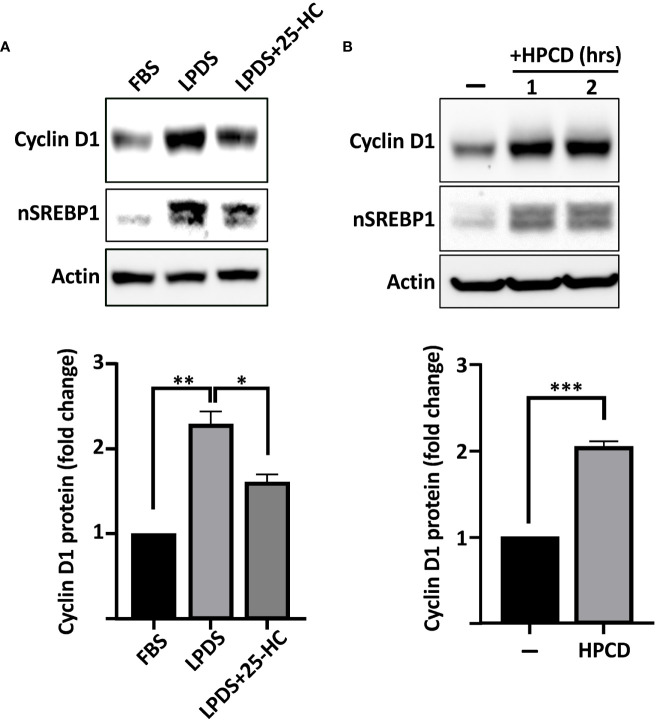
The expression of cyclin D1 responds to physiological changes in SREBP activity. **(A)** HepG2 cells were grown in media supplemented with regular serum (*FBS*) or lipoprotein-deficient serum (*LPDS*). Where indicated, the LPDS was supplemented with 25-hydroxycholesterol (*25-HC*, 5 μg/ml) for 8 h before the end of the experiment. The levels of cyclin D1, nuclear SREBP1 (*nSREBP1*), and β-actin were determined by Western blotting (*upper panel*). The relative levels of cyclin D1 were quantified and are presented as fold change (*lower panel*). **(B)** HepG2 cells grown in regular media were left untreated or treated with 1% HPCD (w/v) for the indicated times. The levels of cyclin D1, nuclear SREBP1 (*nSREBP1*), and β-actin were determined by Western blotting (*upper panel*). The relative levels of cyclin D1 in untreated cells (*−*) and cells exposed to HPCD for 2 h (*HPCD*) were quantified and are presented as fold change (*lower panel*). Significance was determined by paired t-tests **(B)** or one-way ANOVA with Tukey’s multiple comparisons adjustment **(A)**. p-values lower than 0.05 were considered statistically significant. *p < 0.05, **p < 0.01, ***p < 0.001, and ****p < 0.0001. *NS*, not significant.

The SREBPs are regulated by intracellular cholesterol levels, where high levels of cholesterol inhibits while cholesterol deficiency activates the transcription factors. To test if the expression of cyclin D1 was sensitive to changes in intracellular sterol levels, wild-type HepG2 cells were grown in either regular media (FBS, high sterols) or media in which FBS was replaced by lipoprotein-deficient serum (LPDS, low sterols). As illustrated in **Figure 2A**, the expression of cyclin D1 protein was increased in cells grown in lipoprotein-deficient serum. Importantly, the induction of cyclin D1 was reversed when the LPDS-containing media was supplemented with 25-hydroxycholesterol (25-HC), a very efficient inhibitor of SREBP activation. Similarly, the levels of cyclin D1 protein was rapidly induced in cells treated with 2-hydroxypropyl-β-cyclodextrin (HPCD) (**Figure 2B**), a compound that extracts cholesterol from the plasma membrane, thereby drastically reducing cellular cholesterol levels. Taken together, these results suggest that the SREBP pathway is a positive regulator of cyclin D1.

### The human cyclin D1 promoter responds to SREBP

**Figure 3:**
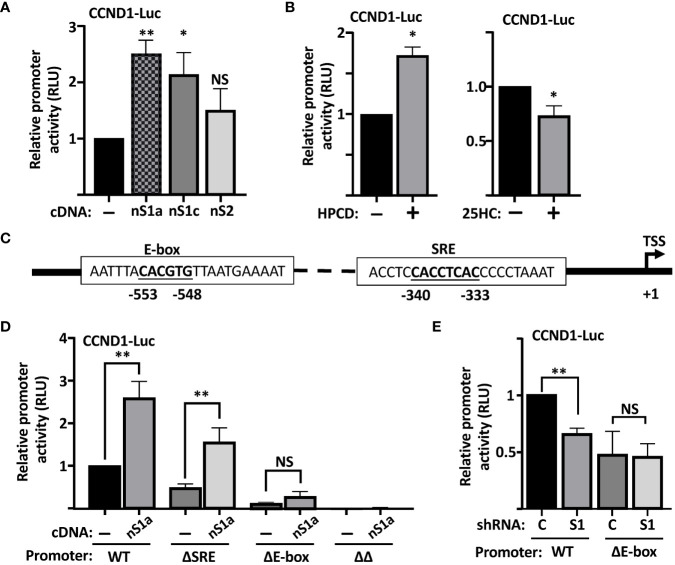
The human cyclin D1 promoter is SREBP-responsive. **(A)** HEK293 cells were transfected with the CCND1-luc promoter-reporter gene (*CCND1-Luc*) together with either empty vector (*pcDNA3*) or expression vectors for the nuclear forms of SREBP1a (*nS1a*), SREBP1c (*nS1c*), or SREBP2 (*nS2*). Forty-eight hours after transfection, cells were lysed, and luciferase activity was measured. **(B)** HepG2 cells were transfected with the CCND1-luc promoter-reporter gene and treated as indicated. Cells in regular media were either left untreated or treated with 1% HPCD (w/v) for 4 h (*left*). Cells in LPDS were either left untreated or treated with 25HC (5 μg/ml) for 8 h (*right*). Forty-eight hours after transfection, cells were lysed, and luciferase activity was measured. **(C)** Schematic illustration of the human cyclin D1 promoter with the location of the E-box and putative SRE elements. **(D)** HepG2 cells were transfected with the CCND1-luc promoter–reporter gene, either wild type (*WT*) or the indicated deletion mutant (*ΔSRE*, *ΔE-box* or *ΔΔ*) together with either empty vector (*pcDNA3*) or an expression vector for the nuclear form of SREBP1a (*nS1a*). Forty-eight hours after transfection, cells were lysed, and luciferase activity was measured. **(E)** HepG2 cells were transfected with the CCND1-luc promoter–reporter gene, either wild type (*WT*) or the ΔE-box mutant, together with expression vectors for either non-targeted **(C)** or SREBP1-targeted (*S1*) shRNA. Forty-eight hours after transfection, cells were lysed, and luciferase activity was measured. Significance was determined by paired t-tests **(B, D, E)** or one-way ANOVA with Tukey’s multiple comparisons adjustment *(A)*. p-values lower than 0.05 were considered statistically significant. *p < 0.05, **p < 0.01, ***p < 0.001, and ****p< 0.0001. *NS*, not significant.

To test if the cyclin D1 promoter is responsive to SREBPs, we used a reporter construct in which the proximal promoter of human cyclin D1 drives the expression of the luciferase reporter gene. The activity of the reporter construct was significantly enhanced when cotransfected with the nuclear forms of SREBP1a and SREBP1c, while the response to SREBP2 failed to reach significance (**Figure 3A**), suggesting that the proximal region of the human cyclin D1 promoter contains an SREBP-responsive element. This hypothesis was supported by the observation that the cyclin D1 promoter–reporter construct was activated in cells treated with HPCD and slightly reduced in cells treated with 25-HC (**Figure 3B**). The latter results are very similar to those observed for the expression of cyclin D1 protein under the same conditions (**Figures 2A, B**).

### The human cyclin D1 promoter contains two potential SREBP binding sites

The SREBPs bind to two distinct nucleotide sequences in target promoters to regulate the expression of the corresponding genes. The first of these is what is known as a sterol-responsive element (SRE) and the other is a classical E-Box (CANNTG) (20). On inspection of the human cyclin D1 promoter, we identified one E-box and one potential SRE (**Figure 3C**). To test the functionality of these elements for the SREBP-dependent regulation of the cyclin D1 promoter, we used site-directed mutagenesis to delete both elements, either alone (ΔE and ΔSRE) or in combination (ΔΔ), in the cyclin D1 promoter–reporter construct. The activity of the ΔSRE promoter was slightly reduced compared to the wild-type promoter, while the activities of the ΔE and ΔΔ promoters were significantly and drastically reduced, respectively (**Figure 3D**). Importantly, the wild-type promoter was induced by cotransfected SREBP1a, while SREBP1a failed to induce the activity of the ΔE construct. The ΔSRE construct retained its sensitivity to SREBP1a but did not reach the same level of induction as the wild-type promoter. Furthermore, the activity of the wild-type promoter was reduced when cotransfected with shRNA targeting SREBP1, while the ΔE promoter was insensitive to the loss of SREBP1. Thus, these results suggest that both the SRE and E-box contribute to the SREBP-dependent regulation of the cyclin D1 promoter, but that the E-box could be of greater importance for this regulation. Our results also clearly demonstrate that deletion of both elements drastically reduces the activity of the promoter (ΔΔ in **Figure 3D**).

### SREBPs interact with the cyclin D1 promoter *in vitro* and *in vivo*

**Figure 4:**
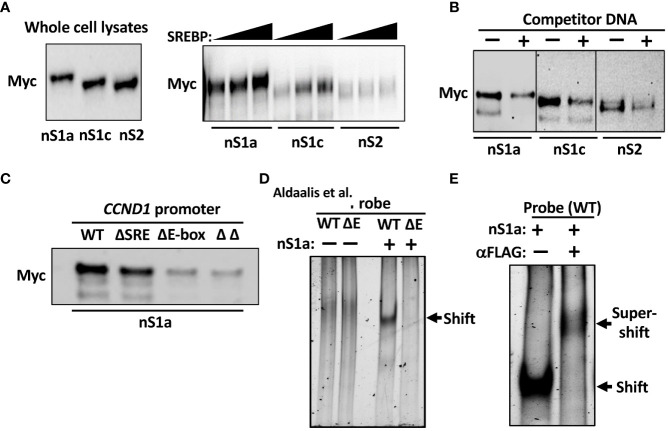
SREBP1 interacts with the human cyclin D1 promoter. **(A)** 6xMyc-tagged nuclear SREBP1a (*nS1a)*, SREBP1c (*nS1c*), and SREBP2 (*nS2*) were expressed in HEK293 cells and used in DNAP assays using a biotinylated DNA probe corresponding to the human cyclin D1 promoter. The three proteins were expressed at similar levels as monitored by Western blotting (*left*). Increasing amounts of the three proteins were incubated with the promoter probe, captured on streptavidin beads, washed, separated on SDS-PAGE gels, and analyzed by Western blotting (*Myc*, *right*). **(B)** Nuclear SREBP1a (*nS1a*, 10 μl), SREBP1c (*nS1c*, 10 μl), and SREBP2 (*nS2*, 50 μl) were incubated with the biotinylated cyclin D1 probe in the absence or presence of non-biotinylated competitor DNA corresponding to the SREBP-binding site in the human LDL receptor promoter (fivefold excess) and processed as in **(A)**. **(C)** Nuclear SREBP1a (*nS1a*) was incubated with the biotinylated cyclin D1 probe, either wild-type (*WT*) or the indicated deletion mutant (*ΔSRE*, *ΔE-box* or *ΔΔ*) and processed as in **(A)**. **(D)** FLAG-tagged nuclear SREBP1a (*nS1a*) was purified from transfected HEK293 cells and incubated with unlabeled DNA probes corresponding to the human cyclin D1 promoter, either wild-type (*WT*) or ΔE-box, separated on native PAGE gels and stained with SYBR Safe. The shifted SREBP1a–DNA complex is indicated by an arrow. The two probes alone were also run on the same gel (*left two lanes*). **(E)** FLAG-tagged nuclear SREBP1a (*nS1a*) was incubated with an unlabeled DNA probe corresponding to the human cyclin D1 promoter, separated on a native PAGE gel, and stained with SYBR Safe. Monoclonal anti-FLAG antibodies were added to the sample in lane 2 prior to loading it on the gel. The shifted SREBP1a–DNA complex and the supershifted SREBP1a–DNA–antibody complexes are indicated by arrows.

Initially, we used DNAP assays to determine if the SREBPs could bind to the cyclin D1 promoter. In these assays, a biotinylated oligo corresponding to the cyclin D1 promoter was used as a bait together with nuclear extracts from cells expressing Myc-tagged nuclear SREBP1a, SREBP1c, or SREBP2. The amount of SREBPs bound to the probe was monitored by Western blotting following capture of the oligos on streptavidin-coated beads. As seen in **Figure 4A**, both SREBP1a and SREBP1c were captured by the cyclin D1 promoter, while the interaction between the promoter and SREBP2 was significantly lower. However, the binding of all three SREBPs to the cyclin D1 promoter probe could be competed out with an excess of an oligo corresponding to the SREBP binding site in the LDL receptor promoter (**Figure 4B**), indicating that these interactions were specific. In these experiments, different amounts of the nuclear SREBPs were used to obtain approximately the same initial binding (see **Figure 4B** legend). To determine if the interaction between SREBP1 with the cyclin D1 promoter was dependent on the SRE or E-box, the experiment was repeated with oligos corresponding to the wild-type promoter or the deletion mutants. As illustrated in **Figure 4C**, the binding between SREBP1a and the cyclin D1 promoter was preserved in the ΔSRE mutant, while deletion of the E-box resulted in a significant reduction in SREBP1a binding. No further reduction in SREBP1a binding was observed in the ΔΔ mutant. These results suggest that the E-box is the dominant SREBP1-binding site in the cyclin D1 promoter, at least *in vitro*.

To confirm these results, we used a separate *in vitro* DNA-binding assay, the EMSA. In this assay, nuclear extracts or purified proteins are incubated with DNA probes and separated on native polyacrylamide gels. The free DNA probe moves fast through the gel, while DNA–protein complexes move more slowly and are shifted upwards in the gel relative to the free probe. We used nuclear SREBP1a purified from transfected HEK293 cells and two separate DNA probes corresponding to the proximal cyclin D1 promoter, either wild-type or the ΔE. As illustrated in **Figure 4D**, nuclear SREBP1a interacted with the wild-type promoter in this assay but was unable to interact with the ΔE promoter, supporting the notion that the E-box is the dominant SREBP1 binding site in the cyclin D1 promoter. However, these results do not exclude the involvement of the potential SRE sequence, especially *in vivo*. To prove that the shifted complex in **Figure 4D** contains SREBP1a, the assay was repeated as before, and an antibody against the FLAG tag in SREBP1a was added to one of the samples prior to loading it on the gel. As seen in **Figure 4E**, the addition of the FLAG antibody shifted the original band even further, reflecting the larger size of the DNA–SREBP1a–antibody complex.

**Figure 5:**
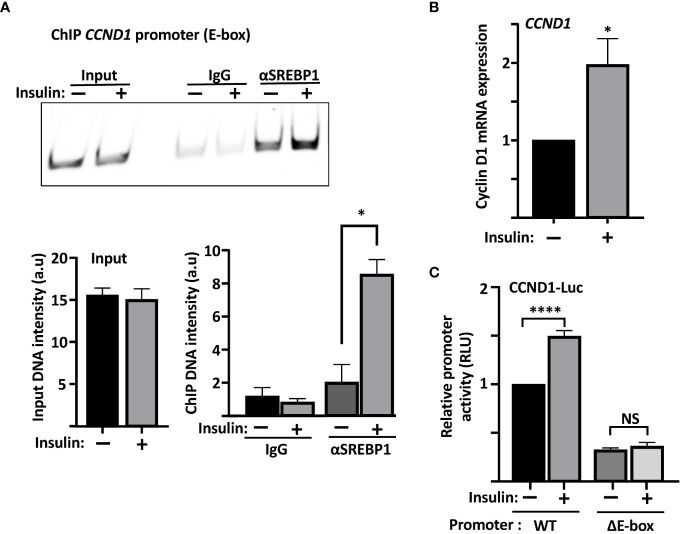
The recruitment of SREBP1 to the cyclin D1 promoter correlates with enhanced expression of the cyclin D1 gene. **(A)** MCF7 cells were serum starved for 24 h and then left untreated or treated with insulin for an additional 2 h. The cells were collected, permeabilized, incubated with SREBP1 or preimmune rabbit *(IgG)* antibodies, followed by the protein A/G-MNase fusion protein. Endogenous SREBP1 and its bound DNA was subsequently isolated using protein A magnetic beads and used for PCR with primers specific for the E-box in the human cyclin D1 promoter. The same primers were also used for PCR of the same region from genomic DNA isolated from the same cells (*Input*). The PCR products were separated on PAGE gels and stained with SYBR Safe (*upper panel*). The intensity of the signals in the samples and the input were quantified (*lower panel*). **(B)** mRNA was isolated from the cells in *(A)* and used to determine the expression of cyclin D1 by real-time qPCR, using GAPDH as a reference. **(C)** MCF7 cells were transfected with the CCND1-luc promoter–reporter gene, either wild-type (*WT*) or the ΔE-box, serum starved for 24 h followed by an additional 2-h incubation in the absence or presence of insulin, after which the cells were lysed, and luciferase activity was measured. Significance was determined by paired t-tests **(A–C)**. p-values lower than 0.05 were considered statistically significant. *p < 0.05, **p < 0.01, ***p < 0.001, and ****p< 0.0001. *NS*, not significant.

Our data so far suggest that SREBP1 is able to bind to the E-box in the cyclin D1 promoter *in vitro*. We decided to use a variety of the ChIP assays, Cut&Run, to determine if this was also true for endogenous SREBP1 in MCF7 cells. As illustrated in **Figure 5A**, an antibody targeting SREBP1 (both SREBP1a and SREBP1c) pulled down DNA sequences corresponding to the E-box in the endogenous cyclin D1 promoter. Importantly, the interaction between SREBP1 and the E-box was significantly enhanced when the MCF7 cells were treated with insulin, a potent activator of SREBP1. The insulin-dependent increase in the binding of SREBP1 to the cyclin D1 promoter correlated to the increased expression of cyclin D1 seen in the same cells (**Figure 5B**). Although we could detect binding of SREBP1 to the SRE as well, the binding was not increased in response to insulin treatment (**Supplementary Figure 4**). Thus, we propose that SREBP1 binds to the E-box in the proximal cyclin D1 promoter and thereby contributes to the insulin-dependent expression of the cyclin D1 gene. This hypothesis was supported by our observation that the wild-type cyclin D1 promoter-reporter gene was induced by insulin in MCF7 cells, while the ΔE construct was not (**Figure 5C**).

### Inactivation of SREBP1 affects the proliferation of MCF7 cells

**Figure 6:**
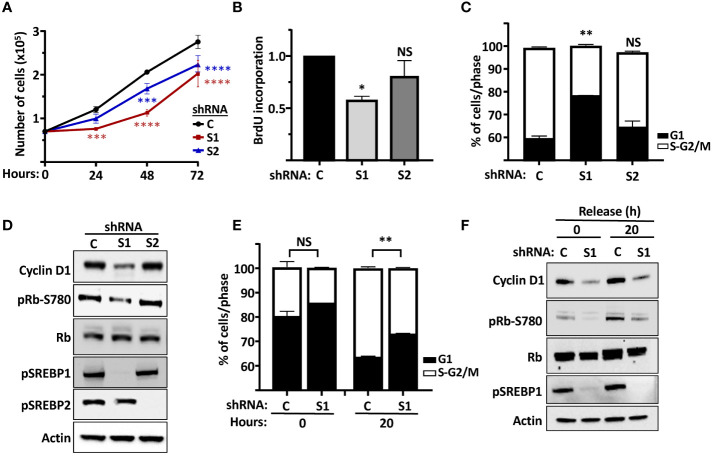
The SREBP pathway regulates the proliferation of MCF7 cells. **(A)** MCF7 cells were transduced with lentiviruses expressing non-targeted shRNA **(C)** or shRNA targeting SREBP1 (*S1*) or SREBP2 *(S2)*. After selection, an equal number of cells were seeded in 12-well plates and cells counted over a 72-h period. **(B)** MCF7 cells were transduced with lentiviruses expressing non-targeted shRNA **(C)** or shRNA targeting SREBP1 (*S1*) or SREBP2 (*S2*) and metabolically labeled with BrdU, and the incorporation of BrdU was determined by an ELISA assay. **(C)** MCF7 cells were transduced with lentiviruses expressing non-targeted shRNA **(C)** or shRNA targeting SREBP1 (*S1*) or SREBP2 (*S2*). The cells were collected, fixed, and stained with propidium iodine, and the DNA content was analyzed by FACS. **(D)** The same cells as in **(C)** were lysed and the levels of cyclin D1, Rb, and the precursor form of SREBP1 (*pSREBP1*), and the phosphorylation of Rb on serine 780 (*pRb-S780*) were analyzed by Western blotting. β-Actin was used as loading control. **(E)** MCF7 cells were transduced with lentiviruses expressing non-targeted shRNA **(C)** or shRNA targeting SREBP1 (*S1*). After selection, the cells were serum-starved for 24 h, followed by an additional 20-h incubation in the absence or presence of serum. The cells were collected and processed as in **(C)**. **(F)** The same cells as in **(E)** were lysed and the levels of cyclin D1, Rb and the precursor form of SREBP1 (*pSREBP1*), and the phosphorylation of Rb on serine 780 (*pRb-S780*) were analyzed by Western blotting. β-Actin was used as loading control. Significance was determined by paired t-tests *(E)* or one-way ANOVA with Tukey’s multiple comparisons adjustment **(A–C)**. p-values lower than 0.05 were considered statistically significant. *p < 0.05, **p < 0.01, ***p < 0.001, and ****p < 0.0001. *NS*, not significant.

Our results so far suggest that both SREBP1 and SREBP2 could regulate the expression of cyclin D1, in part by directly interacting with an E-box in the cyclin D1 promoter. In general, we have observed greater effects on the expression of cyclin D1 when both SREBP1 and SREBP2 are knocked down simultaneously. However, we were concerned that the loss of both SREBP proteins could have effects on cell growth beyond the regulation of cyclin D1, since there is a clear need for lipid synthesis to support cell growth. Thus, we decided to focus more on SREBP1 in our cell proliferation studies. One reason to choose SREBP1 is the fact that the activation of SREBP1c is controlled by several growth factors, including insulin. Another reason is that rapidly growing cells, including cancer cells, express high levels of SREBP1a, a strong transactivator of fatty acid, cholesterol, and triglyceride synthesis. Knocking down both SREBP1 and SREBP2 individually in asynchronous MCF7 cells resulted in a significant reduction in cell growth when cell numbers were monitored over time (**Figure 6A**). Knocking down SREBP1 in asynchronous MCF7 cells also reduced the incorporation of BrdU (DNA synthesis, S phase), while the reduction observed in SREBP2-deficient cells failed to reach significance (**Figure 6B**). Similar results were obtained following FACS analysis of asynchronous MCF7 cells. The SREBP1-deficient cells displayed a higher number of cells in G1 compared to control cells (**Figure 6C**). A similar trend was observed in SREBP2-deficient cells, but the effect just barely reached significance. Importantly, the expression of cyclin D1 protein was reduced in the SREBP1-deficient cells, and this was accompanied by a reduction in Rb phosphorylation (**Figure 6D**), the main cell cycle target of cyclin D1-cdk4/6.

The data presented in **Figures 6A–D**, suggested that the SREBP-dependent expression of cyclin D1 could impact on the phosphorylation and inactivation of Rb, something that could explain the slower growth rate of SREBP1/2-deficient cells. To explore this in more detail, wild-type and SREBP1-deficient MCF7 cells were arrested in G1 by serum starvation followed by a 20-h release in serum-containing media. Samples were collected for FACS and Western blot analyses at both time points. As seen in **Figure 6E**, a larger proportion of SREBP1-deficient cells remained arrested in G1 following serum stimulation when compared to control cells (**Figure 6E**). Importantly, this partial G1 arrest correlated with lower expression of cyclin D1 and Rb phosphorylation in these cells (**Figure 6F**). The same results were obtained when control and SREBP1-deficient cells were followed over an extended time after serum stimulation. A larger proportion of cells remained in G1 in the SREBP1-deficient cells, and the expression of cyclin D1 and the phosphorylation of Rb was lower in these same cells at all time points (**Supplementary Figure 5**). Thus, inactivation of SREBP1 and 2 attenuates the proliferation of MCF7 cells, possibly by regulating cyclin D1-dependent phosphorylation of Rb.

### The SREBP-dependent regulation of Rb is dependent on cyclin D1 and cdk4/6

**Figure 7:**
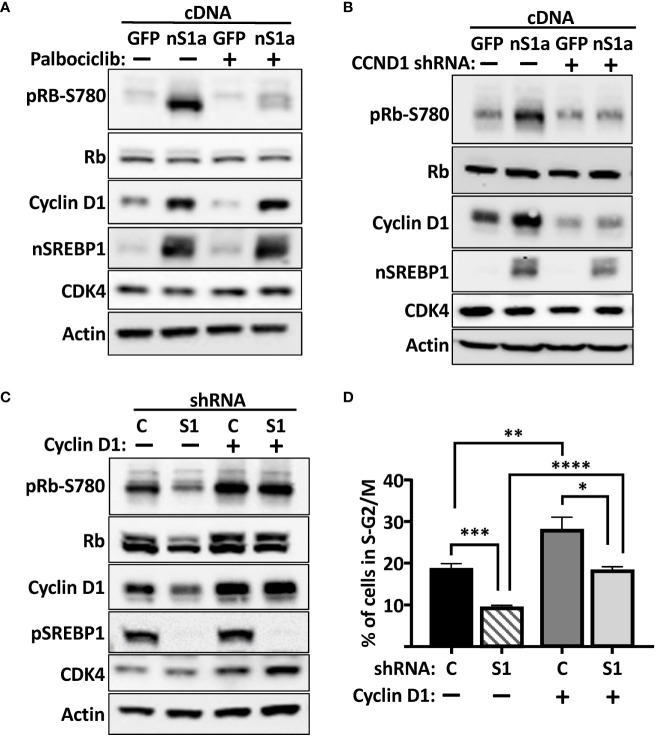
The SREBP1-dependent induction of cyclin D1 promotes the phosphorylation of Rb. **(A)** HepG2 cells were transduced with lentiviruses expressing GFP or nuclear SREBP1a (*nS1a*), and 48 h following transduction, the cells were treated with or without the cdk4/6 inhibitor palbociclib (10 μM) for 6 h. The levels of cyclin D1, Rb, and the nuclear form of SREBP1 (*nSREBP1*), and the phosphorylation of Rb on serine 780 (*pRb-S780*) were analyzed by Western blotting. β-Actin was used as loading control. **(B)** HepG2 cells were transduced with lentiviruses expressing GFP or nuclear SREBP1a (*nS1a*) together with non-targeted **(C)** or cyclin D1 (*CCND1*) shRNA. Forty-eight hours after transduction, the levels of cyclin D1, Rb, CDK4, and nuclear SREBP1 (*nSREBP1*), and the phosphorylation of Rb on serine 780 (*pRb-S780*) were analyzed by Western blotting. β-Actin was used as loading control. **(C)** HepG2 cells were transduced with lentiviruses expressing GFP or cyclin D1 together with non-targeted **(C)** or SREBP1 (*S1*) shRNA. Forty-eight hours after transduction, the levels of cyclin D1, Rb, CDK4 and the precursor form of SREBP1 (*pSREBP1*), and the phosphorylation of Rb on serine 780 (*pRb-S780*) were analyzed by Western blotting. β-Actin was used as loading control. **(D)** The same cells as in **(C)** were also fixed and stained with propidium iodine, and the DNA content was analyzed by FACS. Significance was determined by one-way ANOVA with Tukey’s multiple comparisons adjustment **(D)**. p-values lower than 0.05 were considered statistically significant. *p < 0.05, **p < 0.01, ***p < 0.001, and ****p < 0.0001.

Based on our results, we hypothesized that the SREBP-dependent activation of the cyclin D1 gene should promote the cdk4/6-dependent phosphorylation of Rb, thereby promoting cell proliferation. To test this hypothesis, HepG2 cells were transduced with lentiviruses expressing either GFP (control) or the nuclear form of SREBP1a. Subsequently, the cells were left untreated or treated with the cdk4/6 inhibitor palbociclib for 6 h. As illustrated in **Figure 7A**, expression of nuclear SREBP1a induced the protein levels of cyclin D1 and the phosphorylation of Rb. Importantly, the SREBP1-dependent phosphorylation of Rb was reduced in response to the cdk4/6 inhibitor, suggesting that the phosphorylation was dependent on the kinase activity of cdk4/6. In order to test if the SREBP1-dependent induction of Rb phosphorylation was dependent on cyclin D1, HepG2 cells were transduced with nuclear SREBP1a in the presence of non-targeted (control) or cyclin D1 shRNA. As seen in **Figure 7B**, knockdown of cyclin D1 attenuated the SREBP1-dependent phosphorylation of Rb, suggesting that the SREBP1-dependent induction of cyclin D1 stimulates the phosphorylation of Rb by activating cdk4/6. If this is the case, expression of exogenous cyclin D1 should rescue the phosphorylation of Rb in SREBP1-deficient cells. To test this possibility, HepG2 cells were transduced with non-targeted (control) or SREBP1-targeted shRNAs together with constructs expressing GFP or cyclin D1. As expected, inactivation of SREBP1 resulted in reduced expression of cyclin D1 and decreased phosphorylation of Rb (**Figure 7C**). Importantly, the phosphorylation of Rb was restored in SREBP1-deficient cells expressing exogenous cyclin D1. In agreement with the results obtained in MCF7 cells, the cell cycle profiles indicated that inactivation of SREBP1 in HepG2 cells resulted in a partial G1 arrest (**Figure 7D**). Interestingly, expression of exogenous cyclin D1 normalized the cell cycle profile of the SREBP1-deficient cells, suggesting that the reduced expression of cyclin D1 contributes to the cell cycle disturbances observed in these cells.

**Figure 8:**
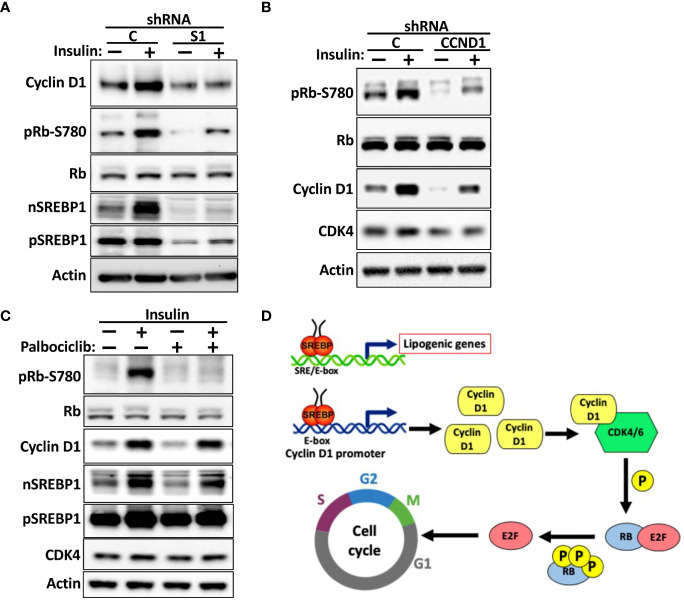
The SREBP1-dependent expression of cyclin D1 links insulin signaling to Rb phosphorylation. **(A)** MCF7 cells were transduced with lentiviruses expressing non-targeted **(C)** or SREBP1 (*S1*) shRNA. After selection, the cells were serum-starved for 24 h, followed by an additional 2-h incubation in the absence or presence of insulin. The levels of cyclin D1, Rb, and nuclear (*nSREBP1*) and precursor SREBP1 (*pSREBP1*) and CDK4, and the phosphorylation of Rb on serine 780 (*pRb-S780*) were analyzed by Western blotting. **(B)** MCF7 cells were transduced with lentiviruses expressing non-targeted **(C)** or cyclin D1 (*CCND1*) shRNA and treated and processed as in **(A)**. **(C)** MCF7 cells were serum-starved for 24 h followed by a 2-h stimulation with insulin in the absence or presence of palbociclib (10 μM). The cells were processed and analyzed as in **(A)**. **(D)** Model. We propose that cells respond to growth-promoting signals, such as insulin or EGF, by activating SREBP1. The active SREBP1 molecules bind to an E-box in the promoter of cyclin D1, thereby enhancing the expression of the corresponding gene. The enhanced expression of cyclin D1 promotes the phosphorylation of Rb by activating cdk4/6, thereby promoting G1 progression. At the same time, SREBP1, especially SREBP1a, has the capacity to enhance the expression of lipogenic genes to support cell growth. Thus, the SREBP-dependent regulation of cyclin D1 could help coordinate cell cycle progression with increased lipid synthesis to support cell growth. This may be especially important in tumor cells, which express the most potent member of the SREBP family of transcription factors, SREBP1a.

Insulin is a major positive regulator of SREBP1c, and the hyperinsulinemia associated with obesity and type 2 diabetes is a risk factor for certain human cancers, including liver and breast cancer (53–56). We (**Figure 5B**) and others have demonstrated that the cyclin D1 gene is induced in response to insulin signaling. To test if SREBP1 plays a role in the insulin-dependent induction of cyclin D1, MCF7 cells were transduced with non-targeted or SREBP1-targeted shRNA and subsequently serum starved for 24 h followed by a 2-h stimulation with insulin. As seen in **Figure 8A**, insulin induced the accumulation of cyclin D1 in control cells, and this response was attenuated in the SREBP1-deficient cells. Insulin also enhanced the phosphorylation of Rb, and this was also attenuated in the SREBP1-deficient cells, suggesting that the insulin-dependent activation of SREBP1 in MCF7 cells results in the induction of cyclin D1, which in turn promotes the phosphorylation of Rb. To test if the insulin-dependent phosphorylation of Rb was dependent on cyclin D1, MCF7 cells were transduced with control or cyclin D1 shRNA and then treated as above. Inactivation of cyclin D1 resulted in a significant reduction in the phosphorylation of Rb (**Figure 8B**). To test if the insulin-dependent phosphorylation of Rb was dependent on the kinase activities of cdk4/6, wild-type MCF7 cells were stimulated with insulin in absence or presence of the cdk4/6 inhibitor palbociclib. As seen in **Figure 8C**, the insulin-dependent phosphorylation of Rb was almost completely blocked in the presence of palbociclib. Thus, our data suggest that the SREBP1-dependent induction of cyclin D1 plays a role in the phosphorylation and inactivation Rb downstream of insulin stimulation, something that could contribute to the growth-promoting effects of this hormone. Taken together, our data support a model in which SREBP1 activates cyclin D1 expression by binding to an E-box in its proximal promoter. The induction of cyclin D1 subsequently promotes the kinase activity of cdk4/6, which in turn results in the phosphorylation and inactivation of Rb and progression through G1 (**Figure 8D**). At the same time, SREBP1, and especially SREBP1a, has the ability to promote the expression of genes involved in *de novo* lipid synthesis. Thus, the SREBP pathway could help coordinate cell proliferation with increased lipid synthesis to support cell growth.

## Discussion

The central regulators of cell cycle progression, the cyclins and their cdk partners, have been studied extensively. Over the last two decades, it has become clear that these same proteins also impact on cellular and organismal metabolism (57–60). This is especially true for cdk4/6 and the D cyclins (61–64). Cdk4 is a target and regulator of insulin signaling in both hepatocytes and adipocytes. In the liver, insulin activates cyclin D1-CDK4, which in turn phosphorylates the histone acetyltransferase GCN5 (65). GCN5 subsequently acetylates peroxisome proliferator-activated receptor gamma coactivator 1-alpha (PGC-1α), which leads to inhibition of the expression of gluconeogenic genes. Thus, activation of cyclin D1-cdk4 suppresses hepatic glucose production in mice in response to feeding. In adipocytes, the cyclin D3–cdk4 complex controls insulin signaling by phosphorylation of the insulin receptor substrate 2 (IRS2), thereby creating a positive feedback loop that maintains adipocyte insulin signaling (66). In addition, both cyclin D3 and cdk4 promote adipogenesis by activating peroxisome-proliferator-activated receptor gamma (PPARγ)-dependent gene regulation (61). Cdk4-deficient mice display impaired insulin signaling and are glucose intolerant. In contrast, mice with hyperactive cdk4 are more glucose tolerant and showed increased insulin sensitivity (61). Thus, cdk4 activity is positively correlated with insulin sensitivity. Crucially, the insulin-dependent induction of cyclin D1 has been shown to be a risk factor for liver cancer in the context of obesity and/or type 2 diabetes (54). Importantly, cyclin D1 overexpression is frequently found in liver and breast cancer (53, 67, 68)

Much less is known about how central regulators of metabolism controls cell cycle progression and thereby cell proliferation. The SREBP family of transcription factors control cholesterol, fatty acid, and triglyceride synthesis and metabolism, and their activities are targets of the statin family of lipid-lowering drugs. Most cells express SREBP1c and SREBP2, where SREBP1c is activated by insulin and mainly controls fatty acid synthesis, while SREBP2 is activated in response to cholesterol deficiency and mainly controls cholesterol synthesis and uptake. The third member of the family, SREBP1a, is restricted to rapidly growing cells, including immune cells, and is the dominant SREBP protein in many tumor cells. One reason for this could be the fact that SREBP1a is a more potent transcription factor compared to SREBP1c and is therefore able to transactivate genes involved in both fatty acid and cholesterol synthesis. Thus, the expression of SREBP1a satisfies the increased demand of lipids to support rapid cell proliferation. Importantly, most tumor cells rely on *de novo* lipid synthesis rather than the external supply of lipids to support their rapid growth. A number of studies have demonstrated that overexpression or loss of SREBP proteins in cancer cells induce and reduce their cell growth, respectively (40, 42, 47, 48, 51, 69–71). In most cases, this has been explained by a deficiency in the synthesis of a particular lipid. However, these observations do not exclude the possibility that the SREBPs could regulate genes directly involved in cell proliferation.

G1 is the only time the cell cycle is responsive to external signals. Thus, the expression and/or function of the cyclins (D, E, and A) and cdks (4, 6, and 2) active during this phase of the cell cycle are targets of both growth promoters and inhibitors. This is especially true for the D cyclins and cdk4/6, since these proteins need to be activated in order to start a new cycle or leave quiescence. The major cell cycle target for the cyclin D-cdk4/6 complex is members of the Rb family of transcriptional repressors. These proteins interact with and inhibit members of the E2F family of proteins (E2F1-3). Cdk4/6-mediated phosphorylation of Rb will reduce its affinity for E2F1-3, thereby enabling them to promote the expression of growth-promoting target genes. Thus, the cyclin D-cdk4/6-Rb-E2F axis is a major regulator of the cell cycle and most human cancers display defects in this signaling pathway, e.g., loss of Rb or amplification of cyclin D and/or cdk4/6. Since the induction of the cyclin D1 gene is an early G1 event, we decided to test if the expression of cyclin D1 was SREBP-responsive. Initial experiments in HepG2 cells indicated that cyclin D1 protein was reduced in response to shRNA-mediated inactivation of SREBP1. The mRNA levels of cyclin D1 in response to SREBP1 inactivation mirrored those seen at the protein level, suggesting that cyclin D1 could be an SREBP1 target gene. Importantly, cyclin D1 protein levels were responsive to changes in cellular cholesterol levels, suggesting that cyclin D1 is responsive to physiological changes in SREBP availability. Cyclin D1 expression was also reduced in MCF7 cells in response to shRNA-mediated inactivation of SREBP1, both at the protein and mRNA levels. Taken together these data suggest that cyclin D1 could be a target for SREBP1, at least in HepG2 and MCF7 cells.

Using promoter–reporter assays, we were able to demonstrate that the human cyclin D1 promoter is responsive to SREBP1. Using *in vitro* and *in vivo* DNA-binding assays we were able to demonstrate that SREBP1 interacts with the cyclin D1 promoter. Our data suggest that SREBP1 binds to an E-box located at position −548 to −553, both after and *in vivo*. The binding of SREBP1 to the E-box was enhanced in response to insulin signaling, which correlated well with the induction of cyclin D1 in the same cells. Importantly, the insulin-responsiveness of the cyclin D1 promoter–reporter gene was dependent on the presence of this E-box. In addition, deletion of the E-box also rendered the cyclin D1 promoter insensitive to SREBP1 loss. These results indicated that SREBP1 could control the insulin-dependent expression of cyclin D1 by binding to an E-box in its promoter. This hypothesis was supported by the observation that the insulin-dependent induction of cyclin D1 was blunted, but not lost, in SREBP1-deficient MCF7 cells. The binding of SREBP1/2 to target promoters is often regulated by other transcription factors binding to neighboring promoter elements, e.g., Sp1 and NF-Y (19, 72). It is possible that such mechanisms could determine the apparent preference of SREBP1 to bind to the E-box in in the cyclin D1 promoter and should be addressed in future studies.

Insulin is an important mitogen for many cell types. It has been demonstrated that cyclin D1 is overexpressed in the liver in response to hyperinsulinemia, a risk factor for the development of liver cancer. Importantly, inactivation of cyclin D1 in the liver drastically reduced the incidence of liver cancer in obese/diabetic mice (54). The expression and activation of SREBP1c is greatly enhanced in response to hyperinsulinemia, primarily as a result of mTORC1 activation. It would therefore be interesting to explore if inactivation/inhibition of SREBP1 would also reduce the incidence of liver cancer under these conditions. Interestingly, it has been shown that cyclin D-cdk4/6 activates mTORC1 in breast cancer cells, including MCF7 cells (63). The authors suggested that the activation of mTORC1 downstream of cyclin D-cdk4/6 is important to couple the cell cycle machinery to cell growth. Considering the important role of mTORC1 in the control of SREBP1, it will be interesting to explore if SREBP1 is activated through this mechanism. The cyclin D1 gene is also induced downstream of other growth factors (1). Many of these factors, including EGF and PDGF, also activate SREBP1 (38, 41, 42). It will, therefore, be important to determine if the SREBP pathway is involved in the regulation of cyclin D1 downstream of other growth-promoting and/or oncogenic pathways. Although we present strong evidence of the involvement of SREBP1/2 in the expression of the cyclin D1 gene, the results do not exclude the possibility that these transcription factors could regulate the expression of cyclin D1 protein in other ways, such as mRNA stability, translational efficiency or protein stability (9–11, 73–76). In addition, inactivation of SREBP1 could potentially regulate cyclin D1 expression indirectly by restricting the supply of lipids for cell growth. However, this cannot explain the recruitment of endogenous SREBP1 to the E-box in the cyclin D1 promoter or the fact that the SREBP-responsiveness of the cyclin D1 promoter-gene is dependent on this same E-box element. However, further studies of the mechanisms involved in the SREBP-dependent regulation of cyclin D1 are warranted.

The main function of cyclin D1 is to activate cdk4/6 and thereby promote the phosphorylation of Rb. In the absence of phosphorylation, Rb inactivates E2F1-3 and prevents the expression of proliferative genes. We were able to demonstrate that the SREBP1-dependent regulation of cyclin D1 affected Rb phosphorylation and cell growth. The phosphorylation of Rb mimicked the changes in cyclin D1 expression following SREBP1 expression (up) or inactivation (down). The expression of cyclin D1 and the phosphorylation of Rb were reduced in asynchronous SREBP1-deficient MCF7 cells, and the cells were partially arrested in G1. We observed the same phenomenon in SREBP1-deficient HepG2 cells. Importantly, both the phosphorylation of Rb and cell cycle arrest could be normalized by the expression of exogenous cyclin D1. However, the number of cells in S-G2/M in the SREBP1-deficient cells expressing exogenous cyclin D1 only reached the level observed in wild-type cells expressing GFP and remained lower than that observed in wild-type cells expressing exogenous cyclin D1. Thus, although the expression of exogenous cyclin D1 restored the phosphorylation of Rb in the SREBP1-deficient cells, it did not fully restore their proliferation. One possibility is that the SREBP1-deficient cells do not have the capacity to synthesize fatty acids and/or cholesterol to fully support proliferation. This possibility will be tested in future experiments by supplementing the SREBP1-deficient cells with specific lipids. Regardless, our results suggest that SREBP1 regulates Rb phosphorylation by regulating cyclin D1 expression. This hypothesis was further supported by the observation that the enhanced phosphorylation of Rb seen in response to SREBP1a expression was dependent on cyclin D1 and cdk4/6. Inhibitors of cdk4/6 are promising cancer therapeutics, and several of these inhibitors are currently used to treat breast cancer. Although initially very effective, the development of resistance to cdk4/6 inhibitors is common. A recent report by Dang et al. demonstrated that Rb is degraded in G1 and that this process is blocked following its phosphorylation by cdk4/6 (14). Consequently, the authors demonstrated that treatment of breast cancer cells with cdk4/6 inhibitors promoted the degradation of Rb. Importantly, the authors found that the expression of Rb was significantly reduced in cells resistant to cdk4/6 inhibitors. The same phenomenon was seen in response to cdk4 and cyclin D1 loss, but interestingly not in response to cyclin D3 loss. Rb inactivation and/or loss is frequently observed in human tumors, and the authors proposed that this could also be an important factor in the development of drug resistance. Their results suggest that cyclin D1–cdk4/6 activity is not only required for the timely inactivation of Rb but also for preventing its degradation. Thus, the activity of this cyclin–cdk complex must be tightly controlled. It will, therefore, be important to determine if the SREBP1-dependent regulation of cyclin D1 affects the stability of Rb.

A recent article reported that the inactivation of SREBP1c in mice resulted in the accumulation of DNA damage, genomic instability, and senescence, specifically in adipose tissue (77). Interestingly, the authors showed that this is probably unrelated to changes in lipid metabolism. Instead, they demonstrated that SREBP1c interacts with PARP, a protein recruited to sites of DNA damage, and promotes its enzymatic activity, thereby sensitizing the DNA damage repair pathway. Of course, senescence is intimately linked to the cell cycle. We did not monitor senescence in our experiments, but it is possible that the loss of SREBP-dependent expression of cyclin D1 could contribute to cell cycle exit and senescence in the long term, such as in SREBP1-deficient mice. However, it is unlikely that the changes in cyclin D1 expression that we report are the result of senescence, since we only inactivated the SREBP genes transiently. In addition, our knockdown cells still proliferated, albeit at a slower rate. However, our work and that reported by Lee et al. clearly illustrate that the SREBP pathway can regulate cell proliferation through mechanisms beyond its ability to enhance lipid synthesis.

Although the main function of cyclin D1 is to activate cdk4/6 and thereby enhance the phosphorylation and inhibition of Rb, other functions have also been ascribed to this cyclin (58, 59). Importantly, cyclin-D1-deficient mice develop hepatic steatosis, possibly because of overactivation of PPARγ (78). This was supported by the observation that the forced overexpression of cyclin D1 in the liver of mice caused a significant downregulation of genes associated with fatty acid synthesis (79). In addition, cyclin D1 has been shown to inhibit the carbohydrate response element-binding protein (ChREBP) (80), a transcription factor that shares many target genes with SREBP1c. Thus, the SREBP-dependent induction of cyclin D1 could be involved in a negative feedback loop to limit lipid synthesis under certain condition. The current study focused on the impact of the SREBP pathway on the canonical function of cyclin D1, and additional work is needed to clarify the full impact of the SREBP-cyclin D1 axis.

Based on the results reported in the current manuscript, we propose that members of the SREBP family of transcription factors promote the expression of cyclin D1, thereby promoting the cdk4/6-mediated phosphorylation and inactivation of Rb. As a result, the expression of cyclin D1 and the phosphorylation of Rb are reduced in SREBP1-deficient cells, resulting in a partial G1 arrest. Thus, the SREBP pathway could contribute to the sustained proliferative phenotype observed in tumor cells, especially since cancer cells express SREBP1a, a very potent transcription factor. Thus, the SREBP1a-dependent regulation of cyclin D1 could help coordinate cell cycle progression with the increased synthesis of lipids needed to support rapid cell growth. Hyperphosphorylation of Rb activates E2F1-3 and thereby the expression of their target genes. If our hypothesis is correct, inactivation of SREBPs should result in a reduced expression of these genes, while overexpression of SREBPs should induce the expression of these same genes. Our future studies will be designed to test this hypothesis. Interestingly, SREBP1 has been identified as target gene for E2F1 (81). Importantly, a recent report demonstrated that the E2F1-dependent induction of SREBP1 plays an important role in supporting the increased demand for lipid synthesis in prostate cancer cells (82). Consequently, inactivation of SREBP1 reduced the proliferation of prostate cancer cells *in vitro* and inhibited tumor growth in animal models. Thus, SREBP1 may not only be a regulator of the cyclin D-cdk4/6-Rb-E2F signaling axis but could also be an important target for this pathway. Future studies are needed to determine if the E2F1-dependent induction of SREBP1 contributes to the expression of cyclin D1 and the inhibition of Rb in tumor cells. However, the current manuscript supports the notion that the SREBP pathway is required for sustained proliferation, both as an inducer of lipid synthesis and as a regulator of cyclin D1, a key driver of cell cycle progression.

## Data availability statement

The raw data supporting the conclusions of this article will be made available by the authors, without undue reservation.

## Author contributions

AA and JE conceptualized the study. AA and MB-A conducted experiments and performed data analysis and manuscript writing. JE secured funding, designed the research, and performed data analysis and manuscript writing. All authors contributed to the article and approved the submitted version.

## Funding

This study was supported by the Qatar National Research Fund (NPRP13S-0127-200178).

## Acknowledgments

We thank all members of the Ericsson laboratory for their feedback and the College of Health and Life Sciences at HBKU for intramural support. Open Access funding provided by the Qatar National Library.

## Conflict of interest

The authors declare that the research was conducted in the absence of any commercial or financial relationships that could be construed as a potential conflict of interest.

## Publisher’s note

All claims expressed in this article are solely those of the authors and do not necessarily represent those of their affiliated organizations, or those of the publisher, the editors and the reviewers. Any product that may be evaluated in this article, or claim that may be made by its manufacturer, is not guaranteed or endorsed by the publisher.
