# Supplementary material for: The SREBP-dependent regulation of cyclin D1 coordinates cell proliferation and lipid synthesis
Source: Front Oncol. 2022 Aug 24;12:942386. doi: 10.3389/fonc.2022.942386 (PMC9451027; doi:10.3389/fonc.2022.942386)
Supplement: Supplementary file 1 [file DataSheet_1.pdf]

A

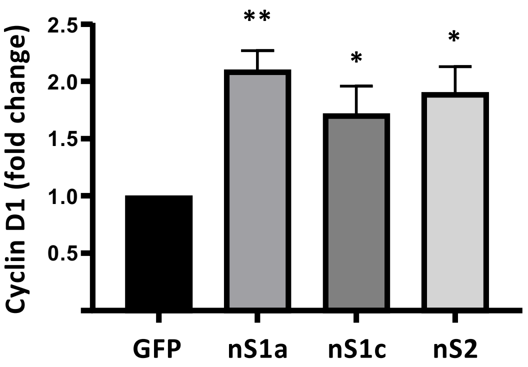

B

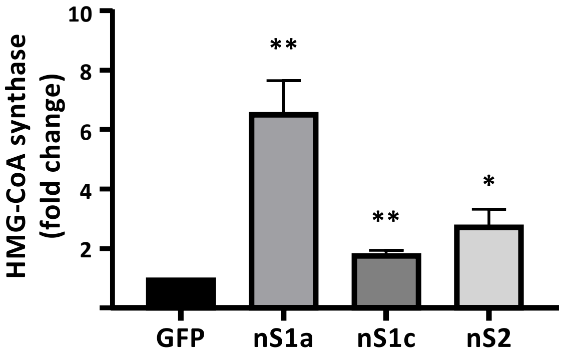

Figure S1. Cyclin D1 is regulated by members of the SREBP family of transcription factors. HepG2 cells were transduced with lentiviruses encoding GFP or FLAG-tagged versions of the nuclear forms of SREBP1a (*nS1a*), SREBP1c (*nS1c*) or SREBP2 (*nS2*). The levels of cyclin D1, HMG-CoA synthase, and β-actin in total lysates were determined by Western blotting and band intensity was quantified to determine the fold change in protein expression. The data represent the averages +/- SEM of at least three independent experiments. Significance was determined by one-way ANOVA with Tukey's multiple comparisons adjustment. *P < 0 .05, **P < 0 .01, ***P < 0 .001, and ****P < 0.0001. Related to Fig. 1A.

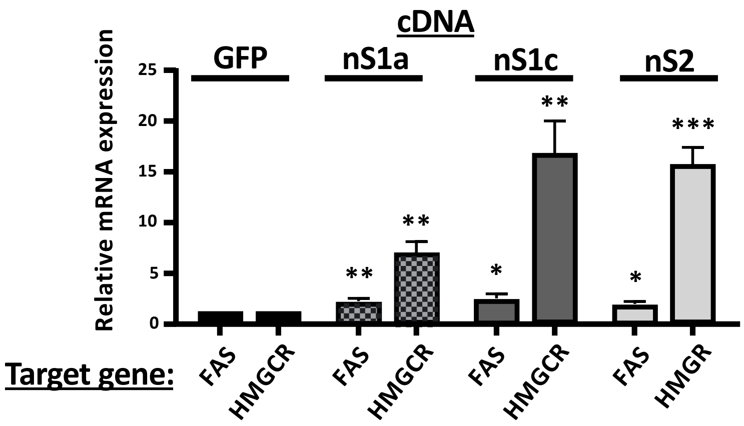

Figure S2. The nuclear forms of SREBP1 and SREBP2 regulate the expression of fatty acid synthase and HMG-CoA reductase. HepG2 cells were transduced with lentiviruses encoding GFP or the nuclear fragments of SREBP1a, SREBP1c or SREBP2 and mRNA was isolated and used to generate cDNA. The expression levels of fatty acid synthase (*FAS*) and HMG-CoA reductase (*HMGCR*) were determined by real-time qPCR using GAPDH for normalization. The data represent the averages +/- SEM of at least three independent experiments. Significance was determined by one-way ANOVA with Tukey's multiple comparisons adjustment. *P < 0 .05, **P < 0 .01, ***P < 0 .001, and ****P < 0.0001. Related to Fig. 1B.

Supplementary Figure 3
Aldaalis et al.

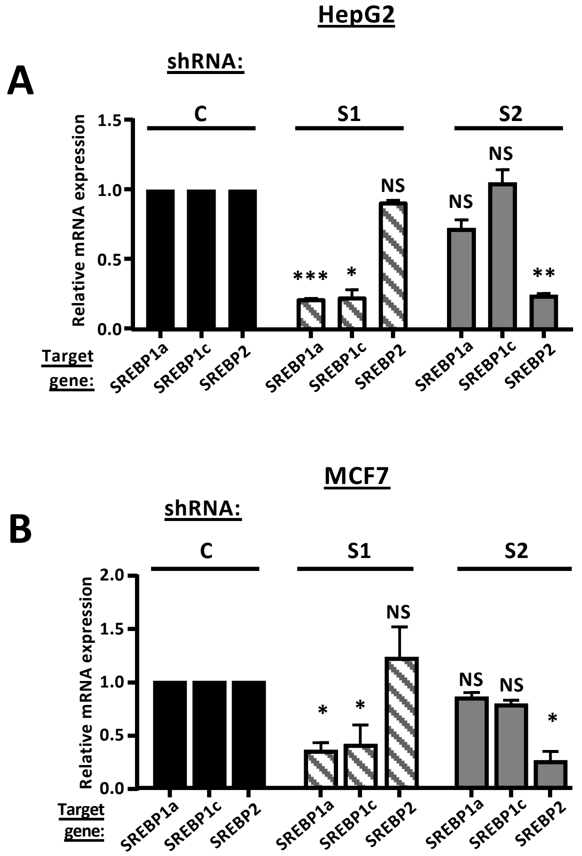

Figure S3. Inactivation of endogenous SREBP1 and SREBP2. (**A**) HepG2 cells were transduced with lentiviruses expressing shRNAs, either non-targeted (*C*) or targeting SREBP1 (*S1*) or SREBP2 (*S2*). The expression levels of SREBP1a, SREBP1c and SREBP2 were determined by real-time qPCR using GAPDH for normalization. (**B**) MCF7 cells were transduced with shRNA as in (*A*) and the expression levels of SREBP1a, SREBP1c and SREBP2 were determined by real-time qPCR using GAPDH for normalization. The data represent the averages +/- SEM of at least three independent experiments. Significance was determined by one-way ANOVA with Tukey's multiple comparisons adjustment. *P < 0 .05, **P < 0 .01, ***P < 0 .001, and ****P < 0.0001. *NS*, not significant. Related to Figs. 1C-F.

ChIP *CCND1* promoter (SRE)

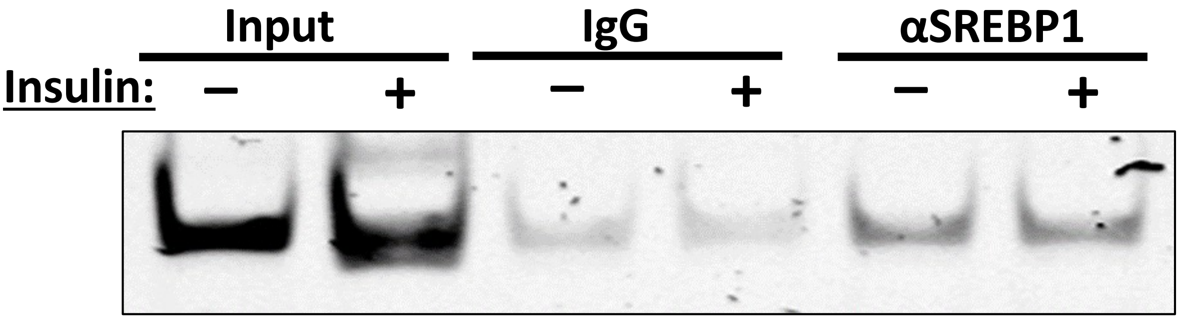

Figure S4. Endogenous SREBP1 interacts weakly with the putative SRE in the human cyclin D1 promoter. MCF7 cells were serum starved for 24 hours and then left untreated or treated with insulin for an additional 2 hours. The cells were collected, permeabilized, incubated with SREBP1 or rabbit preimmune (IgG) antibodies followed by the protein A/G-MNase fusion protein. Endogenous SREBP1 and its bound DNA was subsequently isolated using protein A magnetic beads and used for PCR with primers specific for the putative SRE in the human cyclin D1 promoter. The same primers were also used to PCR up the same region from genomic DNA isolated from the same cells (*Input*). The PCR products were separated on PAGE gels and stained with SYBR Safe. Related to Fig. 5.

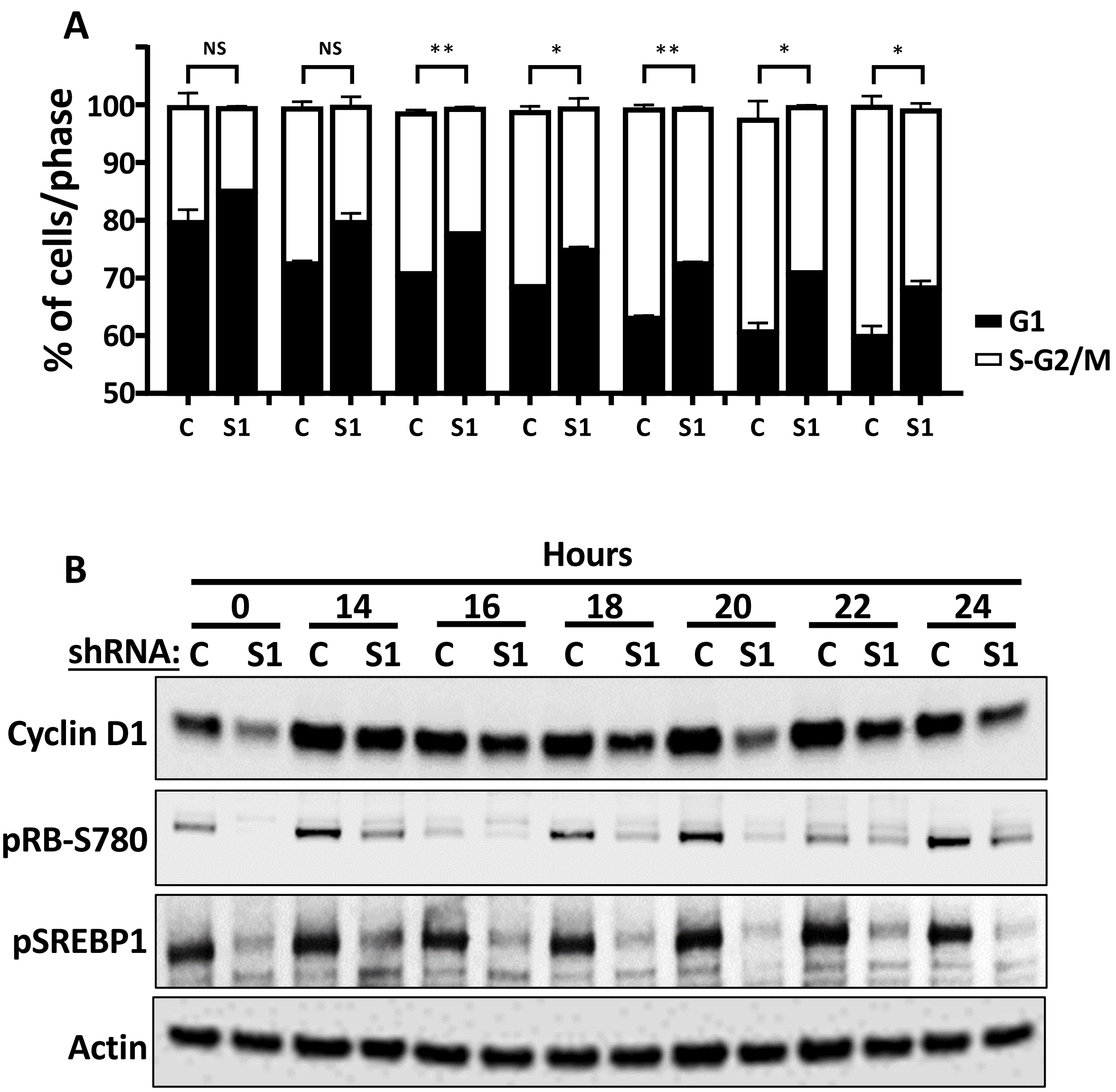

Figure S5. SREBP1 regulates the proliferation of MCF7 cells. (**A**) MCF7 cells were transduced with lentiviruses expressing non-targeted shRNA (*C*) or shRNA targeting SREBP1 (*S1*). After selection, the cells were serum-starved for 24 hours, followed by the addition of serum (10%). The cells were collected at the indicated times after serum addition, fixed and stained with propidium iodine, and the DNA content was analyzed by FACS. The data represent the averages +/- SEM of at least three independent experiments. Significance was determined by paired t-tests. *P < 0 .05, **P < 0 .01, ***P < 0 .001, and ****P < 0.0001. *NS*, not significant. (**B**) The same cells as in (*A*) were lysed and the levels of cyclin D1, precursor SREBP1 (*pSREBP1*), and the phosphorylation of Rb on serine 780 (*pRb-S780*) were analyzed by Western blotting. β-Actin was used as loading control. Related to Figs. 6E-F.
